# Metachronous common iliac lymph node metastasis after rectosigmoid colon cancer resection: A case report

**DOI:** 10.1016/j.ijscr.2021.106203

**Published:** 2021-07-15

**Authors:** Daishi Naoi, Koji Koinuma, Katsusuke Mori, Hisanaga Horie, Alan Kawarai Lefor, Naohiro Sata

**Affiliations:** Department of Surgery, Division of Gastroenterological, General and Transplant Surgery, Jichi Medical University, 3311-1 Yakushiji, Shimotsuke, Tochigi 329-0498, Japan

**Keywords:** CT, computed tomography, 18FDG-PET, F-18 fluorodeoxyglucose positron emission tomography, Colon cancer, Laparoscopic surgery, Lateral lymph node, Case report

## Abstract

**Introduction and importance:**

Metastases to common iliac lymph nodes from cancer of the rectosigmoid are extremely rare. We report a patient with a right common iliac lymph node metastasis after rectosigmoid cancer resection.

**Case presentation:**

The patient is a 57-year-old woman diagnosed with rectosigmoid cancer (Stage IIIc) who underwent laparoscopic resection followed by 8 courses of adjuvant chemotherapy with capecitabine. Sixteen months after resection, an intra-abdominal mass and a left lung nodule were found on computed tomography scans, which were suspected to be recurrences. Exploratory laparoscopy showed that the abdominal lesion was an enlarged common iliac lymph node, which was completely excised. No other intraabdominal recurrences were found. Subsequently, a left upper lobe lung metastasis was resected thoracoscopically. However, multiple lung metastases developed four months after the lung resection, and systemic therapy was begun.

**Clinical discussion:**

A lower incidence of lateral lymph node metastases from cancer in the rectosigmoid has been reported. Direct lymphatic pathways from the sigmoid colon or rectosigmoid to lateral lymph nodes have been suspected, which may be associated with the poor prognosis in this patient.

**Conclusion:**

A metachronous metastasis to a common iliac lymph node from primary rectosigmoid cancer is reported. Common iliac lymph node metastases from rectosigmoid cancer might have more malignant potential, and should be treated in the same manner as peri-aortic lymph node metastases.

## Highlights

• Metastases to common iliac lymph nodes from rectosigmoid cancer are extremely rare.
• Direct spread from the rectosigmoid to a common iliac lymph node is suspected.
• The lymph node metastasis was excised laparoscopically.
• A direct lymphatic pathway may be associated with a poor prognosis.

## 1 Introduction

The presence of lymph node metastases in patients with colorectal cancer is an important prognostic factor. Therapeutic guidelines from the TNM Committee of the International Union Against Cancer are based on the distribution and absolute number of metastatic lymph nodes [1]. The main route of lymphatic drainage of the rectosigmoid is along the inferior mesenteric artery, and metastases to lateral lymph nodes are rare.

In this patient, an isolated common iliac lymph node recurrence without other lateral lymph node metastases was found 16 months after laparoscopic rectosigmoid cancer resection. A lung metastasis was detected simultaneously and both metastatic lesions were resected at a tertiary care University Hospital.

This work is reported in accordance with the SCARE criteria [2].

## 2 Case presentation

**Fig. 1:**
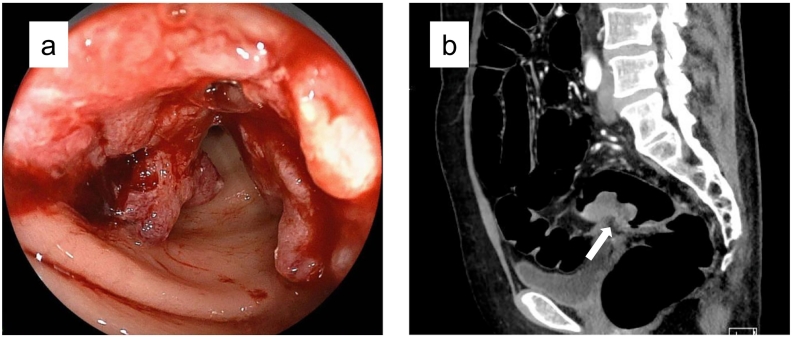
Rectosigmoid colon cancer (arrow) was diagnosed by colonoscopy (a) and computed tomography (b) scan sixteen months before presentation and treated with laparoscopic high anterior resection.

A 57-year-old woman was diagnosed with rectosigmoid cancer and treated with laparoscopic resection (Fig. 1). Histopathological findings showed moderately differentiated tubular adenocarcinoma with invasion to the serosa (T4a). Eight out of 56 regional lymph nodes were positive for metastases (N2b), pathological stage IIIC. The patient received adjuvant chemotherapy with 8 courses of capecitabine.

**Fig. 2:**
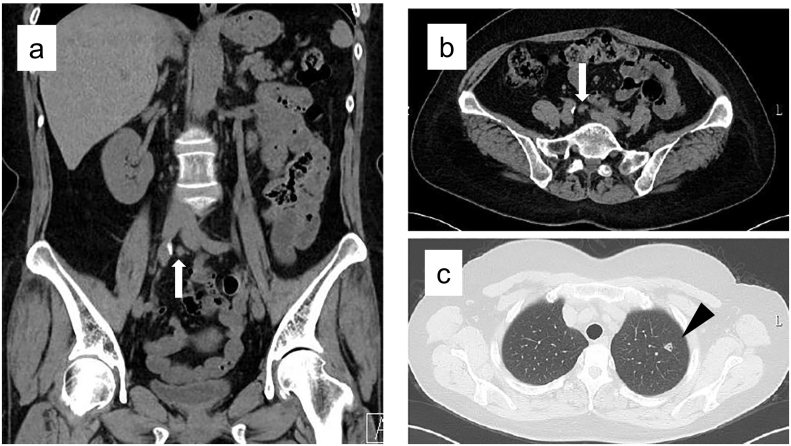
Abdominal computed tomography scan showed an 8 mm nodule (arrow) on the caudal side of the right common iliac artery (a, b), and an 8 mm nodule (arrowhead) in the left upper lobe of the lung (c).

**Fig. 3:**
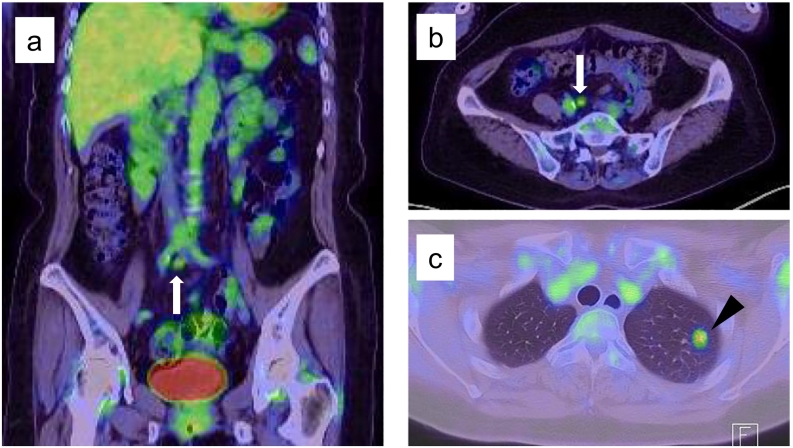
F-18 fluorodeoxyglucose positron emission tomography scan showed high accumulation of fluorodeoxyglucose in both nodules (a, b, c).

**Fig. 4:**
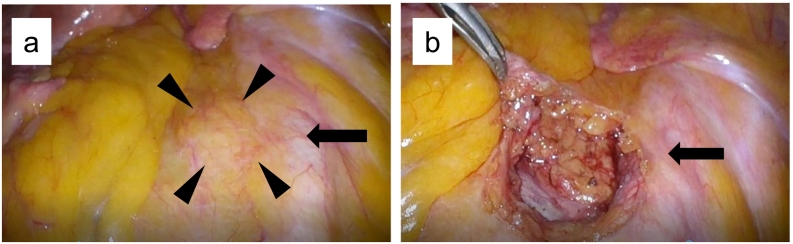
Exploratory laparoscopy revealed a right common iliac lymph node metastasis (arrowhead) (a), and laparoscopic resection was performed (b). Arrow: right common iliac artery.

Sixteen months after resection, an 8 mm-sized nodule was found on the caudal side of the right common iliac artery on follow-up computed tomography (CT) scan. A small nodule was also detected in the left upper lobe of the lung (Fig. 2). Physical examination and laboratory tests including tumor marker values were normal. The F-18 fluorodeoxyglucose positron emission tomography (^18^FDG-PET)/CT scan revealed high accumulations of FDG in the two nodules (intraabdominal and lung) (Fig. 3). The nodule in the pelvis was suspected to be peritoneal dissemination, because lymph node metastases in the common iliac region are extremely rare in patients with rectosigmoid cancer. No other recurrence was detected, and excision was planned. At laparoscopy, no peritoneal dissemination was observed. The nodule observed on ^18^FDG-PET/CT scan was an enlarged lymph node adjacent to the right common iliac artery (Fig. 4). No other lesions were found in the abdomen, and the metastatic lymph node was excised laparoscopically. The post-operative course was uneventful. The histopathological findings confirmed the presence of metastatic tumor in the lymph node consistent with the previously resected rectosigmoid cancer. Subsequently, the upper left lung metastasis was resected thoracoscopically. Four months after the lung resection, multiple lung metastases were found and systemic therapy was begun.

## 3 Discussion

The main route of lymphatic drainage from the rectum is along the superior rectal artery to the inferior mesenteric artery. The lateral route, which reaches from the middle rectal artery to the internal iliac and obturator basins is thought to be an alternative lymphatic drainage pathway [3]. In the Japanese guideline for the treatment of rectal cancer, lateral lymph node dissection is recommended for patients whose rectal tumors are located distal to the peritoneal reflection with the clinical diagnoses of >T3 or >N1 disease preoperatively, because lateral lymph node dissection contributes to a reduction in the local recurrence rate [4], [5]. Lateral lymph nodes are classified into internal iliac, obturator, common iliac, external iliac, median sacral, and aortic bifurcation lymph nodes [6]. Dissection of lateral lymph nodes from the internal iliac and obturator areas contributed to improved survival of patients with lower rectal cancer. Lymph node dissection in these main areas (internal iliac and obturator) was determined to be a part of the standard lateral lymph node dissection in many recent studies describing the surgical treatment of patients with lower rectal cancer [7], [8], [9], [10]. On the contrary, lymph node metastases to other areas including the common iliac lymph nodes were extremely rare, and the index of therapeutic value of lymph node dissection in these areas was almost zero [11].

**Fig. 5:**
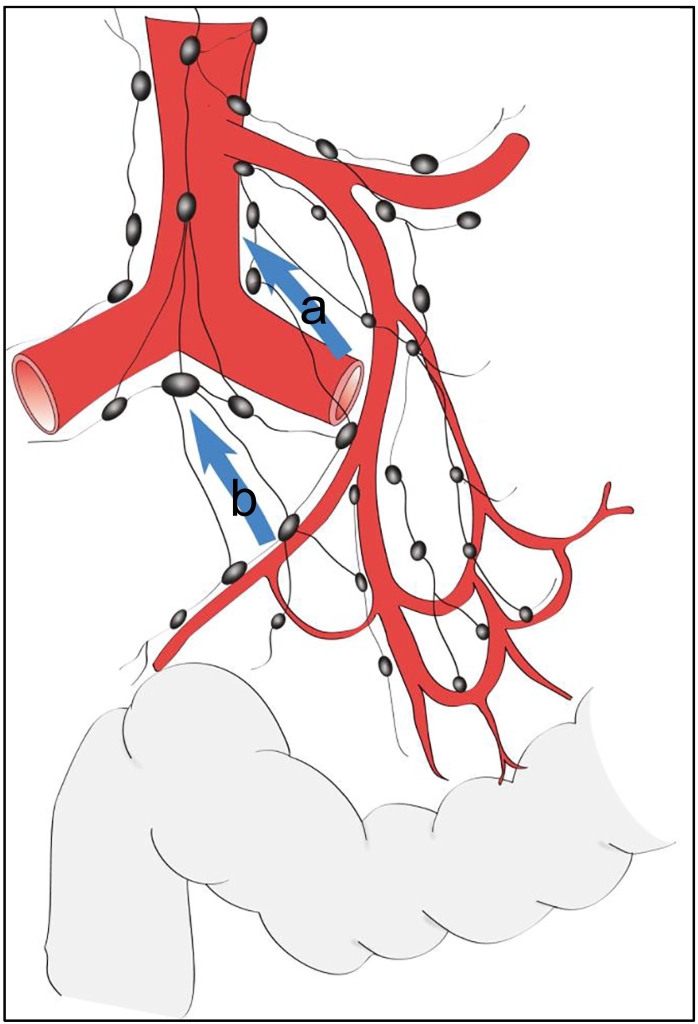
A direct lymphatic pathway providing drainage without passing through the root of the mesenteric artery was described by Jamieson & Dobson (a) [14] and Machiki et al. (b) [15].

Morikawa et al. reported the distribution of metastatic lymph nodes in patients with colorectal cancer, which showed a lower incidence of lateral lymph node metastases from tumors originating in the rectosigmoid [12]. Isolated common iliac lymph node metastases from cancers other than lower rectal cancers are extremely rare. To the best of our knowledge, there is only one report of a case of common iliac lymph node recurrence two years after surgery to resect rectosigmoid cancer. The recurrent lymph node was removed and he had been free of recurrence six years after the second surgery [13]. Normally, lymphatic drainage pathways from colorectal cancer run along with the vessels supplying the tumor. However, alternative lymphatic pathways have been also reported [14], [15]. Jamieson and Dobson reported the existence of lymphatic pathways that flow directly from the vicinity of the colon to para-aortic lymph nodes without passing through the root of the mesenteric artery by examining autopsy samples [14] (Fig. 5a). Machiki et al. reported a patient with sigmoid colon cancer and a metastasis to lymph nodes at the aortic bifurcation. They speculated on the presence of direct lymphatic drainage from the sigmoid colon to the aortic bifurcation lymph nodes [15] (Fig. 5b). The present patient, with an isolated common iliac lymph node recurrence from rectosigmoid cancer, might have developed the metastasis due to the presence of this rare lymphatic pathway. This hypothesis might be supported by the observation that intermediate to main lymph nodes were negative for cancer at the previous rectosigmoid resection, although peri-rectal lymph nodes were positive. Since no liver metastases developed during treatment, it was thought that lung metastases from the rectosigmoid cancer originated in the iliac lymph node and then to the vena cava. Although extremely rare, this lymphatic route of metastasis of rectosigmoid cancer should be considered to guide postoperative surveillance.

(Figure adapted from the description in Refs. [14] and [15].)

The Japanese Society for Cancer of the Colon and Rectum Guidelines 2019 for the treatment of colorectal cancer state that resection of multiple organ metastases can be considered if the recurrent tumors at all sites are resectable [4]. In this patient, both recurrent lesions were considered resectable at the time of diagnosis. However, multiple lung metastases appeared just four months after the lung resection. We suggest that this direct lymphatic pathway may be associated with poorer prognosis in patients with sigmoid or rectosigmoid cancer. Thus, common iliac lymph node metastases from rectosigmoid cancer should be managed as having an inherent progressive aspect similar to peri-aortic lymph node metastases.

## 4 Conclusion

A rare recurrence to a common iliac lymph node from rectosigmoid cancer is reported. The presence of a direct lymphatic drainage pathway from rectosigmoid to common iliac lymph nodes is suspected. Common iliac lymph node metastases from rectosigmoid cancer might have more malignant potential, and should be treated in the same manner as a peri-aortic lymph node metastasis.

## Consent

Written informed consent was obtained from the patient for publication of this case report and accompanying images. A copy of the written consent is available for review by the Editor-in-Chief of this journal on request.

## Provenance and peer review

Not commissioned, externally peer-reviewed.

## Ethical approval

This is a case report and it didn't require ethical approval from ethics committee according to our institution.

## Funding

This research did not receive any specific grant from funding agencies in the public, commercial, or not-for-profit sectors.

## Guarantor

Koji Koinuma.

## Research registration number

Not applicable.

## CRediT authorship contribution statement

Naoi D and Koinuma K: conception of study, acquisition, analysis and interpretation of data.
Katsusuke M and Sadatomo A: analysis of data.
Naoi D, Koinuma K, Ota G, Horie H and Sata N: management of the patient.
Naoi D: Drafting the article.
Koinuma K, Horie H, Lefor A, Sata N: Critical revision of article and final approval of the version to be submitted.

## Declaration of competing interest

All authors declare no conflicts of interest regarding the publication of this paper.
